# Fellowships, Grants, & Awards

**Published:** 2005-05

## Lung Response to Inhaled Highly Toxic Chemicals

The purpose of this program announcement (PA) is to investigate acute mucosal irritation in the upper and lower respiratory tract occurring after aerosol exposure to toxic chemicals with the goals to: 1) minimize initial injury promptly, 2) retard and ameliorate progressive mucosal irritation or inflammation, and 3) offer prophylaxis against pulmonary edema, if created by acute lung injury.

The National Heart, Lung, and Blood Institute (NHLBI) and the NIEHS are concerned about the U.S. population’s potential inhalational exposure to aerosolized harmful chemicals, possibly liberated as part of bioterrorism attacks against assembled groups of the civilian populace. Therefore, research is needed on how humans and relevant animal models respond to inhaled toxic chemicals. The goals of this PA are to develop better bio-protective therapies and to minimize respiratory injury and illness.

Many volatile toxic chemicals are produced and utilized in industry. Some of these are considered hazardous when they are inhaled in ambient air, introduced into food and water supplies, or make contact with body skin surfaces. Among toxic industrial materials that are considered highly hazardous are ammonia, chlorine, formaldehyde, hydrogen cyanine, fuming nitric acid, phosgene, and sulfur dioxide.

From a pulmonary perspective, inhalation exposure to some of these highly hazardous and irritative chemicals induces initial choking, inability to breathe deeply, and excessive output of secretions in the nose and throat from acute irritation. Other chemicals that have neurological effects—including such nerve agents as sarin, certain organophosphate-based pesticides, soman, and others—enter the body through absorption from the airways.

The NHLBI has a limited portfolio of existing research applicable to the respiratory exposures discussed. This PA will stimulate and build research against airborne chemical threats that affect the upper and lower respiratory tract, and will suggest potential therapy to prevent or limit development of pulmonary edema, which is a major complication of airway chemical irritation. Examples of research topics that are of interest include the following: 1) investigating mechanisms of chemical injury (including minimal threshold levels to establish injury) and subsequent effects at a cellular and molecular level causing airway inflammation or hypersensitivity; 2) identifying host responses to initial or immediate effects, and to long-term low-level exposure effects; 3) assessing systematic amount or dose of chemical absorbed from the airways; 4) developing preexposure preventive treatment or early use of antidotes; and 5) devising therapeutic strategies, especially if acute alveolar lung injury occurs and pulmonary edema ensues; specific therapies to prevent onset of pulmonary edema are sought. Development of physical protection (including facial masks and respirators) or environmental detectors for documenting exposure are not within the purview of this announcement.

The NIEHS encourages applications to study chemical exposures relating to civilian terrorism attack, industrial sabotage, or large-scale accidental exposure to toxic chemicals. Applications should focus on research that will develop or support development of treatment strategies that prevent or minimize respiratory track injury following exposure or that maximize repair of injured tissue. To be considered responsive to the NIEHS, the chemical exposure should be acute.

Multiple routes of chemical exposure (respiratory tract, skin, eye, digestive tract) are acceptable if injury resulting from the exposure is specific to the lung. Use of animal models and appropriate human biological specimens is encouraged. Examples of research topics for the NIEHS include but are not limited to the following: 1) the relationship between exposure, route of exposure, and absorbed dose to onset and magnitude of respiratory symptoms in a young, adult, and senior model; 2) cellular and molecular mechanisms of lung injury following acute chemical exposure, including induction of mucosal injury, pulmonary inflammation, acute alveolar injury, and pulmonary edema; 3) cellular and molecular mechanisms of lung tissue repair following acute chemical-induced lung injury; 4) development of postexposure strategies that prevent or minimize lung injury, including early use of antidotes; and 5) development of therapeutic strategies that promote lung tissue repair and that prevent or treat pulmonary edema.

This funding opportunity will use the NIH R01 award mechanism. As an applicant, you will be solely responsible for planning, directing, and executing the proposed project. This funding opportunity uses just-in-time concepts. It also uses the modular as well as the nonmodular budget formats (see http://grants.nih.gov/grants/funding/modular/modular.htm). Specifically, if you are submitting an application with direct costs in each year of $250,000 or less, use the modular budget format described in the PHS 398 application instructions. Otherwise, follow the instructions for nonmodular research grant applications.

Applications must be prepared using the most current PHS 398 research grant application instructions and forms. The PHS 398 application instructions are available at http://grants.nih.gov/grants/funding/phs398/phs398.html in an interactive format. For further assistance contact GrantsInfo at 301-435-0714 or by e-mailing GrantsInfo@nih.gov. Applications must have a Dun & Bradstreet Data Universal Numbering System (DUNS) number as the universal identifier when applying for federal grants or cooperative agreements. This number can be obtained by calling 1-866-705-5711 or through the website at http://www.dnb.com/us/.

Applications must be mailed on or before the receipt date described at http://grants.nih.gov/grants/funding/submissionschedule.htm. The complete version of this PA is available online at http://grants.nih.gov/grants/guide/pa-files/PA-05-058.html.

Contact: Herbert Y. Reynolds, Division of Lung Diseases, NHLBI, 6701 Rockledge Dr, Two Rockledge Ctr, Ste 10018, MSC 7952, Bethesda, MD 20892 USA, 301-435-0218, fax: 301-480-3557, e-mail: Reynoldh@nhlbi.nih.gov; Sally S. Tinkle, Cellular, Organ and Systems Pathobiology Branch, Division of Extramural Research and Training, NIEHS, 111 TW Alexander Dr, PO Box 12233, MD EC-23, Research Triangle Park, NC 27709 USA, 919-541-5327, fax: 919-541-5064, e-mail: tinkle@niehs.nih.gov. Reference PA No. PA-05-058

## *In Utero* Exposure to Bioactive Food Components and Mammary Cancer Risk

*In utero* exposures are important determinants of some cancers occurring in children and young adults. For example, exposure to ionizing radiation *in utero* promotes childhood leukemia, and maternal use of diethylstilbestrol during pregnancy has been linked to clear-cell adenocarcinoma of the vagina in these women’s daughters. In addition, maternal diets—specifically the consumption of vegetables, fruits and protein—are linked to decreased risk of childhood leukemia.

The prenatal period is critical in the development of the mammary gland. During this time, the mammary gland is in a largely undifferentiated state, making it particularly vulnerable to a host of environmental forces. Inappropriate nutritional status or exposure to environmental chemicals and the accompanied alteration in growth and endocrine homeostasis may permanently change the fetus’s structure, physiology, and metabolism, thereby predisposing it to various diseases in later life including mammary cancer.

Epidemiological studies suggest that altering the intrauterine nutritional status can increase mammary cancer risk. Failure of the materno-placental supply line to satisfy fetal nutrient requirements can result in a range of fetal adaptations and developmental changes. Birth weight is a gross surrogate marker for shifts in a host of metabolic processes. Many, but not all, studies reveal a positive relationship between increased birth weight and breast cancer risk. Likewise, other indicators of fetal size such as increased placental weight and birth length are positively correlated with breast cancer risk in the offspring. Recent studies suggest that birth weight is independent from neonatal growth patterns and the timing of puberty as a risk factor for breast cancer.

In addition to nutrition, the hormonal environment in the womb may play an important role in programming lifelong risk for breast cancer in female offspring. A reduction in circulating levels of estrogens and insulin-like growth factor 1 (IGF-1) and/or elevated levels of progesterone, androgens, human chorionic gonadotrophin, IGF-1 binding proteins 1 and 3, cortisol, and insulin have been associated with reduced risk. Such hormonal and growth factor changes are observed during preeclampsia. Maternal preeclampsia has been associated with a reduction in the female offspring’s later risk for breast cancer after adjustment for a variety of potential confounders.

Proliferation of primitive ductal structures in the newborn breast leads to branching and terminal end buds (TEBs). The expansion of TEBs represents an opportunity for malignant transformation because they contain pluripotent mammary stem cells. In fact, *in utero* exposures that bring about an increase in TEBs coincide with increased mammary carcinogenesis. Evidence exists that providing maternal diets that contain elevated amounts of n-6 polyunsaturated fatty acids (PUFAs) and genistein not only increased TEBs but also reduced the differentiation of TEBs to lobuloalveolar units. These diets also increased subsequent chemically induced mammary cancer in the offspring. In addition, prenatal exposures to environmental agents such as bisphenol A or dioxin results in alteration in the development of the mammary gland that may predispose to the development of cancers later in life. Some of this response may relate to changes in hormonal and growth factor status, including status of estrogen and IGF-1.

Greater estrogen exposure throughout a woman’s life has been identified as a major risk factor for the development of breast cancer. *In utero* exposures to the mammary gland can achieve concentrations 10–100 times the estrogen levels occurring later in life. Dietary factors, such as genistein and fat, that influence estrogen exposure to the fetus are related to subsequent cancer risk in several model systems. However, the response may not be totally explained by estradiol, because diets rich in n-3 fatty acids, when fed to pregnant rats, elevate this hormone but reduce mammary cancer incidence in the offspring.

It is possible that intrauterine exposure to other hormones or environmental hormone mimics or antagonists may also affect breast cancer susceptibility. Androgen exposure *in utero* may confer long-term protection against breast cancer by antagonizing the effects of estrogens on fetal breast ductal development. Dietary fatty acids, phytoestrogens, alcohol, and lycopene are among the various bioactive food components reported to influence androgen concentrations. Environmental agents with estrogenic agonist or antagonist activity may also alter gene expression during development, which may lead to functional deficits later in life that predispose one to cancer development. Thus there is the need for studies focusing on uncovering the mechanisms responsible for the protective and detrimental effects on breast cancer risk of exposure to bioactive food components and other environmental agents *in utero*. These studies should attempt to more comprehensively address the changes in all potentially relevant pregnancy hormones and growth factors.

Although the effects of *in utero* exposure to dietary components have been inadequately examined, considerable evidence exists for their ability to modify IGF-1 concentrations and mammary cancer susceptibility postnatally. Postnatal caloric restriction decreases IGF-1 and decreases mammary tumor growth and metastases. Furthermore, postnatal soy phytochemicals combined with green tea synergistically inhibited mammary tumor growth and depressed serum IGF-1 levels in mice. Future studies are warranted to determine whether *in utero* exposure to dietary manipulations that modulate IGF-1 expression will influence subsequent breast cancer risk.

Maternal nutritional status can also alter the epigenetic state of the fetal genome and imprint gene expression levels with lifelong consequences. Loss of imprinting is the silencing of active imprinted genes or the activation of silent imprinted genes, and is one of the most common epigenetic changes associated with the development of a wide variety of tumors. Several lines of evidence support the relationship between maternal nutrition and epigenetic changes in their offspring. Epigenetic changes may provide a molecular mechanism for the impact of maternal nutrition or environmental chemical exposures on postnatal disease susceptibility and deserves future research.

Investigators may choose from the full range of preclinical approaches. The use of genetically engineered animal models including transgenic or knockouts, such as those available through the Mouse Models of Human Cancer Consortium (MMHCC, http://emice.nci.nih.gov/), is encouraged. Studies that apply new high-throughput genomic, epigenomic, proteomic, and metabolomic technologies to determine how dietary and/or environmental chemical exposures *in utero* influence adult breast cancer susceptibility are encouraged.

This funding opportunity will use the NIH investigator-initiated research project grants (R01) and exploratory/developmental (R21) award mechanisms. Illustrative examples for the development of R01 or R21 applications include, but are not limited to, the following: 1) utilization of transgenic and knockout mouse models of human mammary cancer to identify molecular sites of action of bioactive food components in cancer prevention; 2) examination of the role of moderate caloric restriction *in utero* on hormone concentrations and mammary cancer prevention; 3) evaluation of synergistic effects of exposure to bioactive food components *in utero* and subsequent mammary cancer risk; 4) evaluation of imprinted genes after exposure to bioactive food components *in utero* and subsequent mammary cancer risk; 5) examination of the role of *in utero* exposures to environmental agents such at mycotoxins, heterocylic amines, bisphenol A, phthalates, and other agents with endocrine-like agonist or antagonist activity and subsequent mammary cancer risk; and 6) examination of the interaction of *in utero* exposures to bioactive food components and exposures to environmental agents in the etiology of breast cancer later in life.

No set-aside funds are available for this funding opportunity. Applicants may request up to 5 years of support for R01 awards with costs appropriately tailored to the proposed work. No limit is set on the costs requested by R01 applicants. An R21 applicant may request a project period of up to 2 years with a combined budget for direct costs of up to $275,000 for the 2-year period. Normally, no more than $200,000 may be requested in any single year.

Because the nature and scope of the proposed research will vary from application to application, it is anticipated that the size and duration of each award will also vary. Although the financial plans of the involved institutes and centers provide support for this program, awards pursuant to this funding opportunity are contingent upon the availability of funds and the receipt of a sufficient number of meritorious applications.

Applications must be prepared using the most current PHS 398 research grant application instructions and forms. The PHS 398 application instructions are available at http://grants.nih.gov/grants/funding/phs398/phs398.html in an interactive format. For further assistance contact GrantsInfo at 301-435-0714 or by e-mailing GrantsInfo@nih.gov. Applications must have a Dun & Bradstreet Data Universal Numbering System (DUNS) number as the universal identifier when applying for federal grants or cooperative agreements. This number can be obtained by calling 1-866-705-5711 or through the website at http://www.dnb.com/us/.

Applications must be received by the dates listed at http://grants.nih.gov/grants/funding/submissionschedule.htm. The complete version of this PA is available at http://grants.nih.gov/grants/guide/pa-files/PA-05-059.html.

Contact: Cindy D. Davis, Division of Cancer Prevention, National Cancer Institute, 6130 Executive Blvd, EPN Rm 3159, MSC 7328, Bethesda, MD 20892-7328 USA, 301-594-9692, fax: 301-480-3925, e-mail: davisci@mail.nih.gov; Mary Frances Picciano, Office of Dietary Supplements, 6100 Executive Blvd, Rm 3B01, Bethesda, MD 20892-7517 USA, 301-435-3608, e-mail: PiccianM@mail.nih.gov; Jerry Heindel, Cellular, Organs, and Systems Pathobiology Branch, Division of Extramural Research and Training, NIEHS, PO Box 12233, Research Triangle Park, NC, 27709 USA, 919-541-0781, fax: 919-541-5064, e-mail: heindelj@niehs.nih.gov. Reference PA No. PA-05-059

